# 411. Characterization of Antibiotic Prophylaxis Prior to Trans Rectal Ultrasound Guided Prostate Needle Biopsy (TRUS PNB): A 5-Year Nationwide Study among Patients in the United States Veterans Health Administration

**DOI:** 10.1093/ofid/ofac492.488

**Published:** 2022-12-15

**Authors:** Jamil Abou Issa, Paola Saroufim, Elie Saade, Curtis Donskey

**Affiliations:** Lebanese American University, Ashrafieh, Beyrouth, Lebanon; Case Western Reserve University, Cleveland, Ohio; Case Western Reserve University, Cleveland, Ohio; Cleveland VA Hospital, Cleveland, Ohio

## Abstract

**Background:**

Prophylactic antibiotics are used prior to TRUS PNB to reduce risk for infectious complications such as UTI, prostatitis, epididymitis, orchitis, bacteremia and sepsis. Fluoroquinolones have been commonly used for prophylaxis, however, the incidence of post TRUS PNB infections caused by fluoroquinolone resistant *Escherichia coli* has increased. The purpose of this study was to characterize the prophylaxis agents used nationwide in the Veterans Health Administration (VHA) prior to TRUS PNB.

**Methods:**

We conducted a review of records of all patients undergoing TRUS PNB in the VHA database from January 1st, 2013 to December 31st, 2018. We collected data about outpatient oral prophylaxis antibiotics, inpatient injectable antibiotics and microbiology results from rectal swabs and urine cultures performed within 90 days prior to the procedure.

**Results:**

Of 153,055 patients undergoing prostate biopsy between January 1st, 2013 and December 31st, 2018, 27.6% (n=42,319) had urine cultures and 3.9% (n=5,294) had rectal swab cultures done within the 90-day window prior to the procedure. Among these who had urine and rectal swabs culture, 3.0% (n=1,292) and 20.1% (n=1,062) were positive for *E. coli*, respectively; 40.2% of *E. coli* isolates recovered from urine and 89.6% recovered from rectal swabs were resistant to fluoroquinolones. Table 1 shows the frequencies and percentages of oral prophylactic agents administered within the 90 days prior to the procedure and injectable antibiotics administered on the day of the procedure.

Frequency and percentage of oral prophylactic agents administered within the 90 days prior to the procedure and injectable antibiotics administered on the day of the procedure

**Figure:**
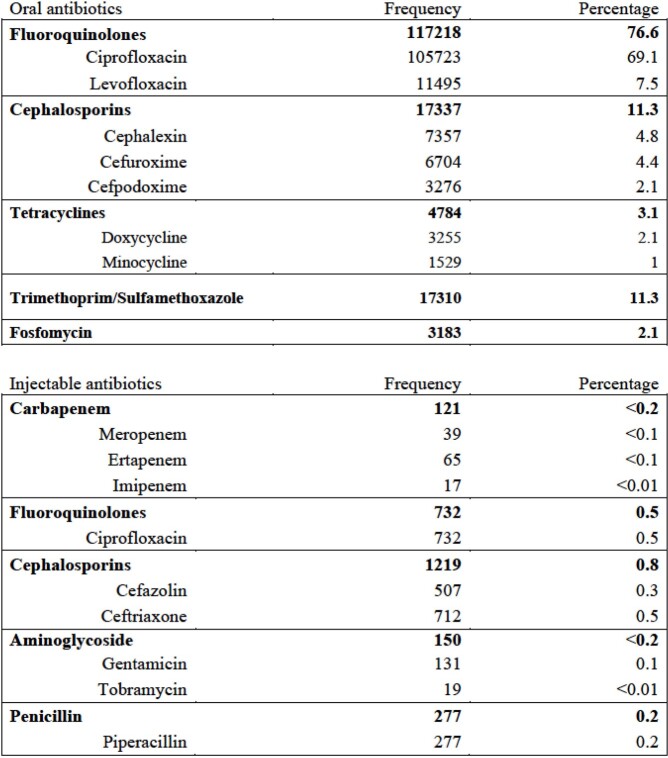

Frequency and percentage of oral prophylactic agents administered within the 90 days prior to the procedure and injectable antibiotics administered on the day of the procedure

**Conclusion:**

Despite the high rate of recovery of fluoroquinolone-resistant *E. coli* in pre-procedure urine and rectal swabs, oral fluoroquinolones remained the most frequently used prophylactic agents prior to TRUS PNB. This inadequate prophylactic coverage may increase the risk of infectious complications following prostate biopsy.

**Disclosures:**

**Elie Saade, MD, MPH, FIDSA**, Janssen: Advisor/Consultant.

